# Functional Outcome, Revision Rates and Mortality after Primary Total Hip Replacement – A National Comparison of Nine Prosthesis Brands in England

**DOI:** 10.1371/journal.pone.0073228

**Published:** 2013-09-04

**Authors:** Mark Pennington, Richard Grieve, Nick Black, Jan H. van der Meulen

**Affiliations:** Department of Health Services Research and Policy, London School of Hygiene and Tropical Medicine, London, United Kingdom; University Medical Center Rotterdam, The Netherlands

## Abstract

**Background:**

The number of prosthesis brands used for hip replacement has increased rapidly, but there is little evidence on their effectiveness. We compared patient-reported outcomes, revision rates, and mortality for the three most frequently used brands within each prosthesis type: cemented (Exeter V40 Contemporary, Exeter V40 Duration and Exeter V40 Elite Plus Ogee), cementless (Corail Pinnacle, Accolade Trident, and Taperloc Exceed), and hybrid (Exeter V40 Trilogy, Exeter V40 Trilogy, and CPT Trilogy).

**Methods and Findings:**

We used three national databases of patients who had hip replacements between 2008 and 2011 in the English NHS to compare functional outcome (Oxford Hip Score (OHS) ranging from 0 (worst) to 48 (best)) in 43,524 patients at six months. We analysed revisions and mortality in 187,201 patients. We used multiple regression to adjust for pre-operative differences. Prosthesis type had an impact on post-operative OHS and revision rates (both p<0.001). Patients with hybrid prostheses had the best functional outcome (mean OHS 39.4, 95%CI 39.1 to 39.7) and those with cemented prostheses the worst (37.7, 37.3 to 38.1). Patients with cemented prostheses had the lowest reported 5-year revision rates (1.3%, 1.2% to 1.4%) and those with cementless prostheses the highest (2.2%, 2.1% to 2.4%). Differences in mortality according to prosthesis type were small and not significant (p = 0.06). Functional outcome varied according to brand among cemented (p = 0.05, with Exeter V40 Duration having the best) and cementless prostheses (p = 0.01, with Corail Pinnacle having the best). Revision rates varied according to brand among hybrids (p = 0.05, with Exeter V40 Trident having the lowest).

**Conclusions:**

Functional outcomes were better with cementless cups and revision rates were lower with cemented stems, which underlies the good overall performance of hybrids. The hybrid Exeter V40 Trident seemed to produce the best overall results. This brand should be considered as a benchmark in randomised trials.

## Introduction

Total hip replacement (THR) has proved to be a highly effective intervention to alleviate pain and improve function in patients with osteoarthritis. The basic technology, a metal stem inserted into the femur and supporting a ball which articulates against a cup inserted into the acetabulum, remains unchanged since its introduction in routine clinical practice in the 1960s [1]. However, cementless attachment for the metal stem, the cup or both components has grown in popularity over fixation with cement [2]. Prostheses with a cementless stem and cup are now the most popular type in many countries [3]–[7].

Four types of prosthesis are being used: cemented (cemented cup and stem), cementless (cementless cup and stem), hybrid (cementless cup and cemented stem), and reverse hybrid (cemented cup and cementless stem). Within each type, there has been a proliferation of different brands (a brand is a distinct combination of a stem and a cup produced by a specific manufacturer). In 2011, 142 brands of femoral stems and 119 brands of acetabular cups were used in the UK [4]. However, the ten most frequently used combinations of stem and cup brands covered nearly half of all THRs undertaken.

Head-to-head randomised controlled trials comparing different types and brands of hip prostheses are rare and trials that exist include brands that have been superseded by newer designs [8]. Consequently, evidence on revision rates according to type and brand comes from non-randomised cohort studies. A study analysing the National Joint Registry of England and Wales (NJR), the largest joint registry in the world, showed that cemented prostheses have a lower revision rate than cementless prostheses but it suggested at the same time an increased risk of mortality in patients who had a cemented prosthesis [9]. A recent economic analysis, also using NJR data, concluded that for most patients hybrid prostheses are the most cost-effective option [10]. It found that cementless prostheses are the most expensive but they do not provide sufficient improvement in health outcomes to justify their additional costs.

The aim of this paper was to compare symptoms, function, quality of life, revision rates and mortality after THR according to prosthesis brand. We did not include reverse hybrid prostheses in the analysis as this type was only used in about 2% of patients who had a THR between 2003 and 2012 in England. We analysed data collected for the National Patient Reported Outcome Measures (PROMs) programme [11]. This programme aims to collect patient characteristics and PROMs immediately before and six months after surgery in all patients undergoing a hip replacement funded by the English National Health Service (NHS). These PROMs data were linked to the NJR and the Hospital Episode Statistics (HES), the administrative database of all admissions to English NHS hospitals [12].

## Methods

### Data sources

The NHS PROMs programme has included patients undergoing a THR funded by the NHS since April 2009 [13]. Patients are invited to complete a questionnaire immediately before surgery and the recruitment rate was 78.8% in the year up to April 2011. Six months after the surgery, a second questionnaire is sent to all patients who completed a pre-operative questionnaire and the response rate was 85.5% in the corresponding period [11].

The pre- and post-operative questionnaires include a generic health-related quality-of-life measure (EQ-5D), and a condition specific measure of symptoms and disability (Oxford Hip Score). The EQ-5D comprises five questions each assessing a specific dimension of health (mobility, self-care, usual activities, pain, and anxiety and depression) with three response levels (“no problems”, “some or moderate problems”, and “extreme problems”) [14]. Responses are converted into a single score on a scale from 1 (perfect health), 0 (death), to −0.596 (worse than death with extreme problems in all five dimensions) [15]. The Oxford Hip Score assesses symptoms and function though 12 items with five response levels. The item scores are summed to generate an overall score that ranges from 0 (worst health status) to 48 (best health status) [16]. The pre-operative questionnaire also asks patients whether they had been told by a doctor that they had any of 12 common serious conditions [17]. The condition “arthritis” was not considered in the analysis as in this study it is the primary disease rather than a comorbid condition.

We obtained the PROMs records of all 178,723 patients who had undergone a THR between April 2008 and June 2011 and who had completed a pre-operative questionnaire. Of these, 108,474 records (60.7%) could be linked to the NJR and HES based on a hierarchical deterministic linkage algorithm at the level of individual patients [11]. This linkage was essential to determine the diagnosis of the hip problem and the prosthesis brand. In addition, it provided information about the patients' socioeconomic status that was derived from their postcode according to the English Index of Multiple Deprivation (IMD) [18]. The IMD ranks 32,482 areas, each of which covers an average population of around 1500 people or 400 households. We grouped the patients into five socioeconomic categories based on fifths of the national ranking of these areas.

We excluded 17,161 patients who were younger than 55 or older than 85 years old, 1589 who received a reverse hybrid prosthesis, 1529 who had a resurfacing procedure, 6749 who had a revision procedure, 4288 who did not have osteoarthritis as the sole diagnosis of their hip problem, 164 who received bone-grafts, 3344 who had minimally invasive surgery, 4 who had a bilateral procedure, 512 privately funded patients, 258 who had a revision within a year of their primary operation, and 329 who died within six months of the surgery. Of the remaining 72,547 patients, we excluded a further 27,322 patients (37.7%) for whom the prosthesis brand was unknown, and 1701 patients whose prosthesis used a metal-on-metal or metal/ceramic combination bearing surface. Of the 43,524 included patients, 16,882 had a cemented, 18,845 cementless, and 7797 a hybrid prosthesis. This “*PROMs data set*” was used to determine quality of life after primary THR.

The NJR collects data on THRs undertaken in England and Wales [19]. Case ascertainment has increased steadily since its inception in 2003. Since 2007, more than 90% of all THRs are being reported to the NJR. Unique patient identifiers allow linkage of primary and revision procedures on the same patient. The data include age, sex, the prosthesis components, American Society of Anesthesiologists (ASA) grade [20], body mass index (BMI), type of hospital (general hospital or specialist joint replacement centre), seniority of operating surgeon (consultant [i.e senior surgeon] or grade below consultant level), funding status of patient (NHS or privately funded), date of revision of the THR, and date of death.

We received from the NJR all “linkable” records of patients aged between 55 and 85 who were reported to have had a unilateral primary THR with a diagnosis of osteoarthritis between April 2003 and March 2012 funded by the NHS (n = 239,012). Linkable records contain the patient's NHS number, a unique patient identifier used throughout the English NHS. The NHS number was used by the NJR Centre to link all primary and revision procedures relating to a single patient.

We excluded 5155 who received a reverse hybrid prosthesis, 14,255 who had a resurfacing procedure, 849 who had a revision rather than a primary THR, 250 patients with missing data on prostheses, 6250 who did not have osteoarthritis as the sole diagnosis, 8329 who received bonegrafts, 5542 who had minimally invasive surgery, and 11,181 who received a prosthesis in which at least one of the bearing surfaces was metal. Of the 187,201 included patients, 91,966 had a cemented, 63,222 a cementless and 32,013 a hybrid, THR. We used the Royal College of Surgeons of England's Charlson Score to determine the number of comorbid conditions (0, 1, or 2 or more) that was captured in HES records of hospital admissions in the year preceding the hip replacement surgery related to 14 major conditions [21]. This “*NJR data set*” was used to determine revision rates and mortality.

We received all data from the Health and Social Care Information Centre in an anonymised format. Given that these data were previously collected in the course of normal care and that the patients were not identifiable to the research team, review by a Research Ethics Committee was not required.

### Missing data

A post-operative PROM questionnaire was missing for 12.2% of patients in the PROMs data set. Moreover, completed post-operative questionnaires contained a small amount of missing or incomplete data for the Oxford Hip Score (1.7%) and a larger corresponding proportion for the EQ-5D (6.9%). To reduce the risk of bias arising from missing PROMS data, we imputed missing items using multiple imputation [22].

In the NJR data set, BMI was missing for 59.4% of patients. Inspection of Kaplan-Meier curves of prosthesis revision rates revealed a profile for patients with missing BMI very similar to those with a BMI of less than 30 kg/m^2^. Consequently, patients with missing BMI data were assumed to have a BMI less than 30. Also, the number of comorbidities according to the Charlson Score was not available for 31.4% of patients and for these patients we added as a separate category.

### Comparisons

Patient-reported outcomes, revision rates and mortality were compared according to the three most commonly used types (cemented, cementless, and hybrid) and according to three most commonly used brands within each type. The frequency with which the types and brands were used was reported in patients operated between April 2009 to June 2012 (cemented: Exeter V40 Contemporary, Exeter V40 Duration, Exeter V40 Elite plus Ogee; cementless: Corail Pinnacle, Accolade Trident, Taperloc Exceed; hybrid: Exeter V40 Trident, Exeter V40 Trilogy, CPT trilogy). The cementless Furlong HAC CSF plus brand was more frequently used (n = 7,531) than the Taperloc Exceed (n = 3,100). There is however less than five years of follow-up data available for the former and therefore it was not included in the comparison as we would not be able to establish the 5-year revision rate.

### Statistical analysis

Linear regression was used to adjust the comparison of post-operative Oxford Hip Score and EQ-5D scores for age, sex, socio-economic status, BMI, ASA grade, number of patient-reported comorbidities, surgeon grade (consultant or not), and hospital type (general or orthopaedic hospital) as well as the pre-operative Oxford Hip Score and EQ-5D scores. Hip replacements of all patients included in the PROMs data set were funded by the NHS and therefore funding status was not included in this regression model. Fractional polynomials were used to represent potential non-linear relationships between the outcome and the factors included in the regression model as continuous variables (age, pre-operative Oxford Hip Score and EQ-5D, socio-economic status, BMI, ASA grade, number of comorbidities) [23]. Overall differences according to the three prosthesis types and according to the three brands within each type were tested with Wald tests and pairwise differences with t-tests.

Cox regression was use to compare revision rates according to prosthesis type and brand with death treated as a censoring event. Differences were expressed as hazard ratios (HR) which can be considered as measures of relative risk. The hazard ratios were adjusted for age, sex, BMI, ASA grade, surgeon grade, hospital type, Charlson score and date of surgery. Overall differences as well as pairwise differences in revision rates according to type and brand were tested with likelihood ratio tests.

Cox regression was also used to compare mortality. We treated revision as a censoring event, since revision operations carry a risk of mortality and we wished to examine whether the primary operation was linked to patient mortality irrespective of the durability of the prosthesis. We adjusted for the same risk factors as used in the comparison of revision rates.

Results are reported with 95% confidence intervals (CI). All hazard ratios and reported p values are based on statistical tests with adjustment for the specified pre-operative characteristics, unless otherwise stated. P values less than 0.05 were considered to indicate statistically significant differences. We did not adjust p values for multiple comparisons. All analyses were undertaken in Stata version 12 [24].

## Results

### Pre-operative characteristics

Patients who received a cemented prosthesis were on average older and more likely to be female, to live in a socioeconomically deprived area, to report at least two comorbidities, and to have an ASA grade of 3 or higher than patients who received a cementless prosthesis (Table 1). Also, patients with a cemented prosthesis were likely before surgery to report more severe symptoms and disability according to the Oxford Hip Score and a poorer quality of life according to the EQ-5D than those with a cementless prosthesis. Most of the characteristics of those receiving a hybrid lie in between those of the cemented and cementless groups.

**Table 1:** Pre-operative characteristics of patients undergoing a primary hip replacement according to prosthesis type in the PROMs data set.

	Cemented	Cementless	Hybrid	Proportion of missing data
No of patients	16,882	18,845	7,797	
Mean (SD) age (years)	72.6 (6.7)	67.7 (7.2)	70.2 (7.2)	0
Women	35.4%	44.0%	38.4%	0
Socio-economic status:				
in most deprived fifth	19.0%	18.8%	23.6%	1.2%
Two or more patient-reported comorbidities	26.3%	21.9%	24.0%	0
ASA grade 3 or higher	18.7%	12.3%	15.5%	0
Mean (SD) body mass index (kg/m^2^)	28.8 (5.2)	29.3 (5.4)	28.9 (5.4)	34.3%
Hip replacement				
at specialist joint replacement centre	7.2%	10.4%	5.3%	0
by consultant, N(%)	81.6%	86.5%	80.7%	0
Pre-operative				
mean (SD) Oxford Hip Score	17.3 (8.0)	18.2 (8.2)	18.2 (8.2)	0.7%
mean (SD) EQ-5D	0.33 (0.32)	0.36 (0.32)	0.35 (0.32)	5.5%

The pre-operative characteristics according to prosthesis brand followed broadly the same pattern as observed for their type (Table 2). A notable exception is that larger proportions of the cemented Exeter V40 Contemporary, the cementless Corail Pinnacle, and the hybrid Exeter V40 Trident were used in orthopaedic hospitals compared to the other brands within their respective type. Also, the hybrid CPT Trilogy was less often implanted by consultant surgeons.

**Table 2:** Pre-operative characteristics of patients undergoing a primary hip replacement according to prosthesis brand in the PROMs data.

		Cemented			Cementless			Hybrid	
	Exeter V40 Contemp	Exeter V40 Duration	Exeter V40 Elite Plus Ogee	Corail Pinnacle	Accolade Trident	Taperloc Exceed	Exeter V40 Trident	Exeter V40 Trilogy	CPT Trilogy
No of patients	6795	1129	1479	8570	1879	1166	2794	891	872
Mean (SD) age (years)	72.9 (6.5)	72.0 (6.9)	73.0 (6.6)	67.6 (7.2)	67.8 (7.2)	66.8 (7.0)	69.0 (7.5)	69.9 (6.7)	71.2 (7.3)
Women	63.5%	69.2%	61.7%	56.5%	56.5%	57.3%	59.6%	55.1%	64.0%
Socio-economic status:									
in most deprived fifth	18.7%	24.1%	14.1%	21.6%	18.9%	14.4%	24.1%	28.6%	32.2%
Two or more patient-reported comorbidities	26.6%	26.6%	26.6%	21.4%	22.8%	21.4%	23.0%	22.3%	24.7%
ASA grade 3 or higher	20.1%	19.5%	17.2%	11.4%	14.7%	11.4%	16.1%	17.3%	13.4%
Mean (SD) body mass index (kg/m2)	28.8 (5.2)	28.6 (5.4)	28.7 (4.9)	29.2 (5.4)	29.5 (5.5)	29.3 (4.9)	29.1 (5.5)	28.4 (5.8)	28.4 (5.0)
Hip replacement									
at specialist joint replacement centre	6.5%	0.7%	2.6%	17.7%	8.8%	4.3%	6.1%	0.2%	2.9%
by consultant	80.0%	80.7%	79.9%	89.0%	87.5%	89.2%	84.6%	86.9%	59.1%
Pre-operative									
Mean (SD) Oxford Hip Score	17.5 (8.0)	17.8 (8.1)	17.5 (8.0)	18.3 (8.2)	18.5 (8.2)	18.2 (8.0)	18.3 (8.2)	18.3 (7.7)	18.6 (8.6)
Mean (SD) EQ-5D	0.34 (0.32)	0.33 (0.32)	0.33 (0.32)	0.36 (0.32)	0.37 (0.32)	0.36 (0.32)	0.35 (0.32)	0.35 (0.31)	0.36 (0.32)

### Post-operative function and quality of life

Post-operative Oxford Hip Scores and EQ-5D values varied according to prosthesis type (overall p values <0.001). Patients who had received a hybrid prosthesis reported the best post-operative function and quality of life (OHS = 39.4, 95% CI 39.1 to 39.7; EQ-5D = 0.80, 0.79 to 0.81) and patients who received a cemented prosthesis the worst (OHS = 37.7, 37.3 to 38.1; EQ-5D = 0.76, 0.75 to 0.77). Patients with a cementless prosthesis reported intermediate outcomes (OHS = 39.2, 38.8 to 39.5; EQ-5D = 0.79, 0.78 to 0.80).

Table 3 presents the post-operative Oxford Hip Scores and EQ-5D values for the three most commonly used brands within each prosthesis type. The Oxford Hip Scores varied according to brand among the cementless prostheses after adjusting for pre-operative differences (overall p value = 0.01). The Corail Pinnacle (the most frequently used cementless brand) had Oxford HIip Scores which were on average 0.5 higher than the Accolade Trident and Taperloc Exceed (both pairwise p values <0.05).

**Table 3:** Post-operative outcomes of primary hip replacement.

		Cemented			Cementless			Hybrid	
	Exeter V40 Contemp	Exeter V40 Duration	Exeter V40 Elite Plus Ogee	Corail Pinnacle	Accolade Trident	Taperloc Exceed	Exeter V40 Trident	Exeter V40 Trilogy	CPT Trilogy
*PROMs data set*									
No of patients	6795	1129	1479	8570	1879	1166	2794	891	872
Post-operative									
Unadjusted mean OHS	37.9	38.3	37.3	39.8	39.1	39.0	39.3	39.7	39.7
(95% CI)	(37.5 to 38.3)	(37.7 to 38.9)	(36.8 to 37.9)	(39.5 to 40.2)	(38.6 to 39.6)	(38.4 to 39.6)	(38.9 to 39.8)	(39.0 to 40.3)	(39.0 to 40.4)
Adjusted difference	reference	0.3	−0.5	reference	−0.5	−0.6	reference	0.1	0.2
(95%CI)		(−0.3 to 0.8)	(−1.0 to 0.0)		(−1.0 to −0.1)	(−1.1 to 0.0)		(−0.5 to 0.8)	(−0.4 to 0.9)
Significance		p = 0.05			p = 0.01			p = 0.7	
Post-operative									
Unadjusted mean EQ-5D	0.75	0.75	0.75	0.79	0.77	0.78	0.78	0.78	0.80
(95% CI)	(0.74 to 0.76)	(0.74 to 0.77)	(0.73 to 0.77)	(0.78 to 0.80)	(0.75 to 0.79)	(0.76 to 0.80)	(0.77 to 0.80)	(0.76 to 0.80)	(0.77 to 0.82)
Adjusted difference	reference	0.00	0.00	reference	−0.01	0.00	reference	−0.01	0.01
(95%CI)		(−0.02 to 0.01)	(−0.01 to 0.01)		(−0.03 to 0.00)	(−0.02 to 0.01)		(−0.03 to 0.01)	(−0.01 to 0.03)
Significance		p = 0.9			p = 0.2			p = 0.4	
*NJR data set*									
No of patients	23,900	6484	8372	21,902	4906	2951	9884	4102	3572
5-year revision rate*	1.25%	1.44%	1.03%	2.06%	2.86%	1.55%	1.34%	1.55%	1.93%
(95%CI)	(1.06 to 1.46)	(1.14 to 1.83)	(0.80 to 1.32)	(1.78 to 2.39)	(2.16 to 3.79)	(1.04 to 2.32)	(1.06 to 1.69)	(1.16 to 2.06)	(1.41 to 2.65)
Hazard ratio	reference	1.33	0.93	reference	1.27	0.97	reference	1.31	1.56
(95%CI)		(1.03 to 1.75)	(0.71 to 1.23)		(0.99 to 1.63)	(0.67 to 1.40)		(0.92 to 1.84)	
Significance		p = 0.06			p = 0.2			p = 0.05	

Figures are percentages unless otherwise stated.

* unadjusted Kaplan Meier estimate for men and women aged 55 to 84.

There is also some evidence for differences according to brand among the cemented prostheses (overall p = 0.05). With adjustment for pre-operative characteristics, the Oxford Hip Score for the Exeter V40 Contemporary (the most frequently used cemented brand) was on average 0.5 higher than for the Exeter V40 Elite Plus Ogee (pairwise p value = 0.04) and 0.3 lower than for the Exeter V40 Duration but the latter difference was not statistically significant (pairwise p value = 0.4).

The Oxford Hip Scores among the three hybrid brands were very similar and there was little evidence of a difference according to brand (overall p = 0.7).

Within each of the three prosthesis types, the EQ-5D values were similar across brands with adjusted differences of 0.01 or less (Table 3). The overall p values within each of the three prosthesis types were all 0.2 or higher.

### Revision rates

Prosthesis type had a significant impact on 5-year revision rates (overall p value <0.001). The 5-year revision was lowest in patients with a cemented prosthesis (1.28%, 95%CI 1.19% to 1.37%) and highest in those with a cementless prosthesis (2.23%, 2.08% to 2.39%). Patients with a hybrid prosthesis had a 5-year revision rate (1.69%, 1.52% to 1.88%) that was in between those with cemented or cementless prosthesis. Compared to patients with a cemented prosthesis, the adjusted hazard ratios for revision rates were 1.66 (1.51 to 1.83; pairwise p value <0.001) for patients with cementless prostheses, and 1.26 (1.12 to 1.42; pairwise p value <0.001) for patients with hybrid prostheses.

The differences in revision rates according to brand within the three types were relatively small with slight evidence of differences according to brand among the cemented (overall p value = 0.06), cementless prostheses (overall p value 0.2), and hybrid prostheses (overall p value = 0.05) if pre-operative characteristics were taken into account. Within cemented prostheses, the Exeter V40 Duration had a revision rate that was higher than that of the Exeter V40 Contemporary (hazard ratio 1.33; pairwise p value = 0.03). Within the hybrid prostheses, the revision rate of the CPT Trilogy was higher than that of the Exeter V40 Trident (hazard ratio 1.56; pairwise p value = 0.02).

### 5-year mortality

Without adjustment for pre-operative characteristics, mortality within the first five years after hip replacement differed considerably according to prosthesis types. Mortality was highest in patients with a cemented prosthesis (10.8%, 10.54% to 11.06%) and lowest in those with a cementless prosthesis (6.9%, 6.6% to 7.2%; unadjusted hazard ratio 0.62, 0.60 to 0.65) with an intermediate mortality result in patients with a hybrid prosthesis (8.2%, 7.8% to 8.6%; unadjusted hazard ratio 0.73, 0.70 to 0.77)). However, these differences were greatly reduced with adjustment for pre-operative characteristics. Compared to patients who had a cemented prosthesis, adjusted hazard ratios for mortality were 0.95 (0.91 to 1.00) with a cementless and 0.95 (0.90 to 1.00) with a hybrid prosthesis (overall p value = 0.06). There was no statistical significant difference in mortality according to brand within each prosthesis type (overall p value = 0.7 within cemented, 0.7 within cementless, and 0.2 within hybrid prostheses).

## Discussion

### Main findings

Our study demonstrates that patients who received a hybrid prosthesis had the best functional outcomes and patients who received a cemented prosthesis had the lowest revision rates. Patients who had a cementless prosthesis had the highest revision rates and intermediate patient-reported outcomes. Our analyses according to brand identified the hybrid Exeter V40 Trident as having lower revision rates than the other hybrid prostheses and the cementless Corail Pinnacle as having better Oxford Hip Scores than the other cementless prostheses.

The differences in mortality among patients who received different types and brands of hip prosthesis were small. Adjustment for pre-operative characteristics hugely decreased the observed differences. We could only use age, sex, BMI, ASA grade and hospital admission for comorbidity in preceding year to capture potential differences in the patients' pre-operative condition when analysing mortality, and it is therefore likely that with a more extensive characterisation of prognostic factors (e.g. nature and severity of comorbidities), the mortality differences would have fully disappeared.

Given these results, the hybrid Exeter V40 Trident prosthesis produced the best overall results as it combined good post-operative function and quality of life and low revision rates compared to the other frequently used prosthesis brands. However, this conclusion has to be interpreted with caution given the statistical uncertainty in our estimates, the multitude of comparisons that we carried out, and the potential residual bias. Although our results may not be strong enough to guide directly the choice of prosthesis brand for individual patients, we argue that the hybrid Exeter V40 Trident should be considered as a yard stick against which alternative clinical options should be compared.

### Strengths and limitations

This study is based on one of the world's largest databases of patient-reported outcomes and revision rates after hip replacement[13], [25]. Given their national coverage, the data represent outcomes of routine practice rather than outcomes observed in studies carried out only in specialist centres or within the context of a clinical trial. Given the large size of this study, we were able to detect small differences in post-operative function and quality of life, revision rates and mortality. We could furthermore use detailed information about the patients' pre-operative characteristics, either reported by the patients themselves or by their clinicians, to adjust for differences in case mix.

We report a large number of statistical comparisons. As a result, the probability of finding significant differences caused just by the play of chance alone is increased. We decided against using a formal procedure to adjust for multiple comparisons as we feel that reporting the actual p values supports a truthful representation of the strength of the evidence. A further argument against adjustment for multiple comparisons is that it is not obvious how the number of comparisons should be counted [26]. Should we adjust for the number of prosthesis brands within each prosthesis type or for all brands considered? Should we also take into account that we compared four different outcomes?

As mentioned earlier, residual confounding cannot be fully ruled out although its impact on the results of comparing prosthesis brands within type is likely to be relatively small. The pre-operative case mix profiles according to brand within type were rather similar which suggests that the impact of unknown confounding would be limited.

We report functional outcomes measured at six months after surgery. A recently published study on symptoms and disability over a 5-year period following THR found substantial improvement in the first year and on average stable outcomes after the first year [27] which suggest that the differences in symptoms and disability between prosthesis types and brands reported in this study will be sustained for at least the first five years.

The revisions were identified by linking hip replacement procedures carried out on the same hip within the NJR. Additional work carried out by the NJR Centre has demonstrated that between 30% to 50% of revisions may have gone undetected by the NJR between 2003 and 2009 [28], [29]. However, detailed analyses of the pattern of underdetection have demonstrated that there is no apparent clustering within individual surgeons or hospitals [29], which suggests that it is also independent of prosthesis type and brand. If that is the case, then incomplete detection of revisions only affected the precision and not the accuracy of the relative differences in reported revision rates.

The extent of underdetection of revisions may have gradually decreased as a result of improving patient recruitment rates and a more complete recording of patient identifiers [29]. We have therefore included date of surgery in the regression model that was used for risk adjustment to minimise bias potentially linked to changes over time in the frequencies of use of prosthesis types and brands.

### Comparison with other studies

Our revision rates according to brand follow largely the revision rates published in the 9^th^ NJR Annual Report [29]. However, a crucial difference between the results published by the NJR Centre and ours is that we have excluded patients who had a prosthesis with metal-on-metal bearing surfaces. They were excluded because it has been suggested that the use of metal-on-metal bearing surfaces could explain the higher revision rates of cementless prostheses [30]. However, even with these patients being excluded, the higher revision rates of cementless brands remained, demonstrating that the metal-on-metal bearing surface is an unlikely explanation.

A study based on the New Zealand Joint Registry found that patients with poorer Oxford Hip Scores at six months have a higher risk of revision in the next five years [31]. It is important to note that the differences in functional outcome and revision rates that we observed between prosthesis types do not follow the same pattern. Cementless prosthesis brands had the best functional outcomes at six months but also the highest revision rates at five years which suggests that the determinants of functional outcome and revision rates do not fully overlap.

It has been suggested based on an analysis of NJR data that there is “a small but significantly increased risk of death with cemented procedures” [9]. That analysis found that mortality after a hip replacement with cementless hip prostheses was 10% lower than with cemented prostheses. The authors suggested that cementation could drive emboli into the circulation which can lead to respiratory problems. However, a number of responses to this paper pointed to selection bias as the most likely explanation for the observed effect on mortality [32]–[34]. In our analysis, we adjusted not only for age, sex and ASA score but also for BMI, number of comorbidities year of surgery and found that the apparent impact of the use of cement was further reduced. For that reason, it is likely that with an even more complete adjustment for pre-operative case mix differences the mortality differences according to prosthesis type will vanish.

A further argument against cementation as a cause for an increase in mortality in patients with cemented prostheses is that we did not find an increased mortality in patients with hybrid prostheses, in which cementation is used to fix the stem of this prosthesis type to the femoral bone.

### Further implications

The differences in outcomes after hip replacement according to prosthesis type are considerably larger than the difference according brand within each type. Post-operative function and quality of life is better with a cementless cup (i.e. patients with cementless or hybrid prostheses) whereas revision rates are lower with a cemented stem (i.e. patients with cemented or hybrid prostheses) which underlines the good overall performance of the hybrid brands.

Our results demonstrate the importance of linking patient-reported outcomes with data on revisions after hip replacement. Linkage with general practice records might provide further data on physical activity, smoking and alcohol use which would allow more complete adjustment for the impact of these case-mix difference. In addition, a national resource of linked data provides a structure within which future randomised controlled trials can be carried out efficiently measuring patient-reported outcomes as well as revision rates. Our non-randomised study demonstrates that the hybrid Exeter V40 Trident should be used as a benchmark against which other brands should be tested. The results of these trials should be used in economic analyses to explore the tradeoffs between different outcomes and to estimate quality-adjusted life years and costs.
